# Mendelian randomization study on association between grip strength and BMD in different age groups

**DOI:** 10.1007/s00774-024-01519-1

**Published:** 2024-06-17

**Authors:** Yingying Zhu, Kede Chi, Jiaci Wang

**Affiliations:** 1 Department of Geriatric Medicine, Zhongshan Hospital of Traditional Chinese Medicine, Zhong Shan, 528400 Guangdong Province China; 2 Department of Orthopedics, Zhongshan Hospital of Traditional Chinese Medicine, No.3, Kangxin Road, Zhong Shan, 528400 Guangdong Province China

**Keywords:** Grip strength, Osteoporosis risk, Different ages, Mendelian randomization

## Abstract

**Introduction:**

This study aimed to use the Mendelian randomization study method to verify the causal relationship between grip strength and bone mineral density (BMD) in different ages and different parts of the body.

**Materials and methods:**

The analysis was based on pooled data from genome-wide association studies (GWAS). Hand grip strength (right) was used as the exposure variable and total body bone mineral density (BMD) of different age groups was used as the outcome variable. Single-nucleotide polymorphisms highly correlated with exposure variables were used as instrumental variables. The inverse variance weighted (IVW) method was used as the primary analysis method, and the Mendelian randomization Egger (MR-Egger) regression and weighted median methods were used as supplementary evidence for the IVW results. Horizontal pleiotropy and heterogeneity tests were conducted to ensure the stability of the results.

**Results:**

Analyzing the GWAS data on osteoporosis as the outcome variable, the IVW analysis showed that osteoporosis risk was associated with decreased grip strength in the 45–60 age group and the risk of declining lumbar spine BMD was associated with decreased grip strength. However, there was no significant correlation between the risk of osteoporosis in other age groups and changes in grip strength.

**Conclusion:**

A causal relationship exists between decreased grip strength and osteoporosis risk in people aged 45–60 years. The risk of BMD declining in the lumbar spine was associated with reduced grip strength.

## Introduction

Osteoporosis, a prevalent chronic metabolic bone disease [1], is marked by reduced bone mass, deterioration of bone tissue structure, and heightened fracture susceptibility. Research has indicated that among Chinese individuals aged 40, osteoporosis affected 5.0% of males and 20.6% of females [2]. Reduced BMD correlates with higher fracture vulnerability [3]. During the early twentieth century, researchers integrated BMD and clinical risk factors to create a calculator for assessing fracture risk [4].Consequently, evaluating BMD plays a vital role in diagnosing osteoporosis and forecasting fractures.

Sarcopenia is a progressive skeletal muscle disease characterized by accelerated loss of muscle mass and function, leading to adverse outcomes such as falls, functional decline, weakness, and death [5]. It primarily affects the elderly population, with muscle strength declining earlier and faster than other body composition changes [6]. Grip strength measurements are recommended for assessing sarcopenia and physical frailty in older adults due to their significant negative correlation with age [7].

The diagnosis of sarcopenia involves assessing muscle mass, strength, and physical function. Some studies have suggested a potential correlation between grip strength and BMD, indicating that grip strength is a crucial predictor of osteoporosis [8–10]. However, conflicting conclusions arise from a bidirectional MR study which found a clear causal relationship between osteoporosis and sarcopenia; specifically, BMD was associated with muscle mass but not muscle strength [11]. It remains unclear whether the regulation of cellular pathways explains the simultaneous or continuous relationship between skeletal muscle synthesis and bone synthesis, as well as the exact causal relationship between the two.

Due to inconsistent findings in clinical studies on the association between low grip strength and osteoporosis incidence [9, 12, 13], it is believed that this inconsistency may be due to factors related to body, disease, and environment. The occurrence of diseases is influenced by multiple factors, making it challenging for traditional epidemiological studies to distinguish causal associations with the target disease [14]. Mendelian randomization studies can establish a causal link between grip strength and BMD by eliminating confounding factors [15].

Traditional cross-sectional studies cannot establish a true causal association between exposure and outcome due to their inability to determine the order of exposure and disease occurrence or account for confounding factors. Randomized controlled trials (RCTs) face challenges such as medical ethics considerations, strict research conditions, compliance with subjects' requirements, and funding limitations that hinder their implementation. In contrast, Mendelian randomization uses genetic variation as instrumental variables to establish a causal relationship between exposure and outcome [16–18], providing support for the association between muscle strength and osteoporosis risk.

Handgrip strength and bone mineral density (BMD) are influenced by factors such as age, gender, height, and weight. Grip strength tends to decline after 60 years old [19], while BMD changes significantly in women aged 45–49 to 55–59 compared to men who show less reduction except between 65 and 69 years old [20]. Turning 60 is an important milestone for both women and men regarding bone mass change. Previous research suggests that hand grip strength and BMD decline synchronously after the age of 60 but are influenced by different factors. Randomized controlled trials often fail to establish true associations between pathogens and diseases, resulting in false positives. Therefore, this study aims to utilize Mendelian randomization analysis to eliminate external confounding factors and examine the correlation between grip strength and bone density across various age groups (particularly individuals aged 45–60 versus those over 60 years old).

## Materials and methods

This study used grip-related phenotypes as exposure factors and total body BMD in five age groups (0–15 years, 15–30 years, 30–45 years, 45–60 years, and over 60 years), lumbar spine BMD and femoral neck BMD were used as the outcome. Three two-sample MR analyses were performed using genetic summary data from GWAS to evaluate the potential causal relationship between grip strength and BMD in five age groups and different parts of the body. Because Mendelian randomization study is a secondary analysis, the original research dataset was not separated by sex. For the reasons mentioned above, it is not possible to perform stratified analysis by sex.

## Data sources

### Source of muscle strength-related phenotypes

The single-nucleotide polymorphism (SNP) associated with grip strength, total body BMD, lumbar spine BMD, and femoral neck BMD in the GWAS data came from the Integrative Epidemiology Unit (IEU) OpenGWAS project (mrcieu.ac.uk) website. The website was accessed on August 15, 2023, and the study population was from Europe and included both males and females. The BMD data supporting this study's findings are openly available in [PubMed] at 10.1016/j.ajhg.2017.12.005 [21]. The data on hand grip strength that support the findings of this study are openly available in [Open GWAS project] at [http://gwas-api.mrcieu.ac.uk] and in [PubMed] at 10.1038/s41467-021-20918-w [22]. According to the relevant literature [23] of grip strength is measured by hand grip meter. The data of Lumbar spine BMD and Femoral neck BMD that support the findings of this study are openly available in [PubMed] at 10.1038/nature14878 [23]. According to the original text, the BMD was measured by dual-emission X-ray absorptiometry (DXA).

The sample size of the dataset was 335,842, with a total of 10,894,596 SNPs. The total body BMD sample size was 67,358, and its distribution was as follows: There were 11,807 samples in the 0–15 years age group, with 9,351,693 SNPs. The sample size in the 15–30 age group was 4,180 people with 8,509,502 SNPs. The sample size in the 30–45 age group was 10,062 people with 9,656,698 SNPs. The sample size over the age of 60 age group was 18,805 people with 10,304,110 SNPs. The sample size in the 45–60 age group was 22,504 people with 11,932,096 SNPs.

### The conditions for SNP as an instrument variable

At first, instrument variables were highly correlated with the exposure, with *P* < 5 × 10^–8^ as a substantial correlation standard. Second, instrument variables have no direct relationship with outcome but only affect outcomes (no pleiotropy) through exposure, and the non-0 (*P* < 0.05) in this study indicated no genetic pleiotropic [24]; Third, instrumental variables were not associated with unmeasured confounding. Since the SNPs selected by the MR method follow the genetic principle that parental alleles are randomly assigned to offspring, the affected environment and acquired life span have little effect, so the study considered instrumental variables as independent of environmental factors such as socioeconomic and culture.

### SNP screening rules

The SNP screening selected the meaningful SNP from the GWAS summary data of grip strength (*P* < 5 × 10^–8^, the linkage disequilibrium coefficient *R*^2^ was 0.001, and the width of the linkage disequilibrium region was 10,000 kb) to ensure that each SNP was independent of each other and exclude the influence of gene pleiotropy on the result [25]. The selected grip strength-related SNP was extracted from the GWAS summary data of total body BMD at different ages; set the minimum R^2^ > 0.8, and the missing SNP was replaced with an SNP with high linkage, deleting the SNP without an alternative site. The information from the above two datasets was summarized, and the SNP directly related to BMD (*P* < 5 × 10^–8^) was removed.

### Analytical method

**Fig. 1:**
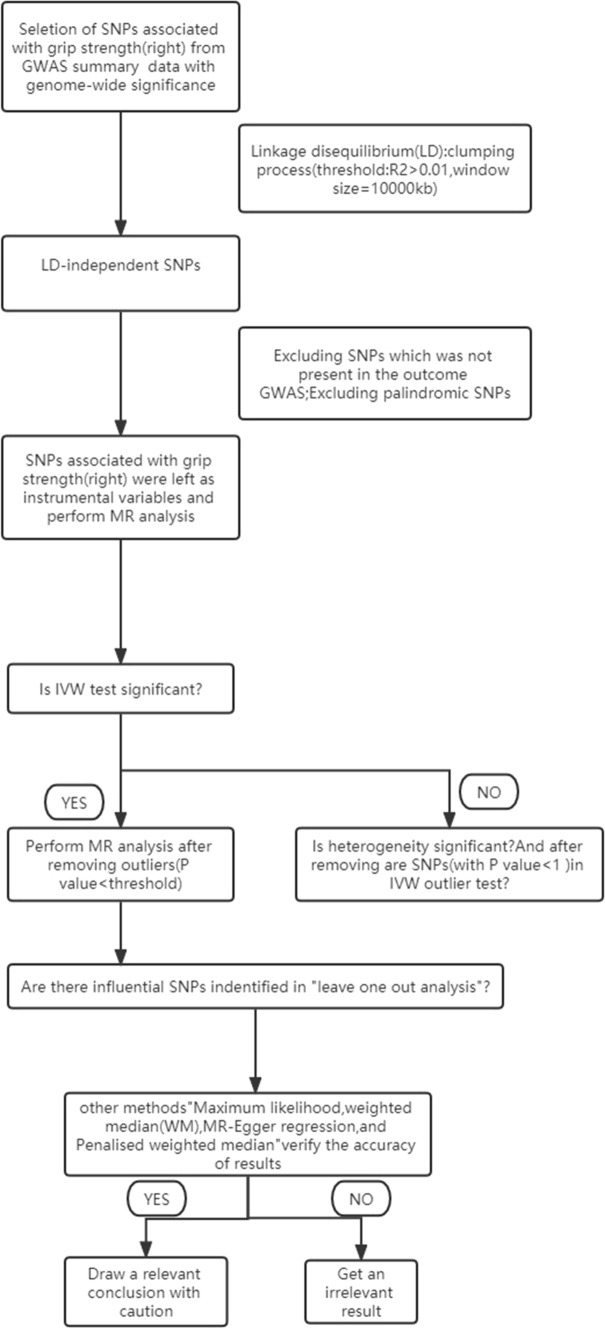
Flowchart

A two-sample MR analysis was performed using the instrumental SNPs to assess the causal effect of grip strength on osteoporosis risk. The summary statistics odd ratio (OR) and standard error of total body BMD at different ages enabled the indirect assessment of the causal association between hand grip strength and osteoporosis (OP) risk. In contrast, pooled comprehensive BMD measures can directly assess OP risk. We also performed the reverse MR analysis. Detailed methods for hand grip strength and BMD analysis included inverse variance weighting (IVW)-random effects, IVW-fixed meta-analysis, maximum likelihood, weighted median (WM), and MR-Egger regression, and the penalized-weighted median was applied to estimate the impact. Bonferroni correction (P-value = 0.05/5 outcomes) was used to adjust for multiple testing (P = 0.005) in this MR. All of these analyses were conducted in R V.4.1.2 using R packages of “Two-Sample MR” (https://mrcieu.github.io/TwoSampleMR/reference/clump_data.html) and P-values < 0.05 were considered statistically significant. The detailed steps are shown in the flowchart (Fig. 1).

The study performed sensitivity analyses to assess whether potential heterogeneity and pleiotropy significantly influence the results. The potential pleiotropy residual sum and outlier were evaluated by MR pleiotropy residual sum and outlier (MR-PRESSO). The Global test was used to detect the presence of heterogeneity and outliers in the instrumental variables (*P* < 0.05), and the Distortion test was used to examine whether there were significant differences in the results before and after removing outliers [26]. MRPRESSO analysis was conducted using the “MRPRESSO” package in R. All analyses were performed in R 4.1.2, and *P* < 0.05 was considered statistically significant.

## Results

### Instrumental variables selected SNP information

After several rounds of screening, a total of 104 grip strength-related SNPs and 514 BMD-related SNPs were included (103 SNPs from 0 to 15 years old, 102 SNPs from 15 to 30 years old, 103 SNPs from 30 to 45 years old, 103 SNPs from 45 to 60 years old, and 103 SNPs from the populations over 60 years old) after preliminary sensitivity analyses. Sixteen SNPs (rs11664336, rs2672591, rs6737767, rs11664336, rs2672591, rs6737767, rs11664336, rs2672591, rs6737767, rs74265413, rs11664336, rs2672591, rs6737767, rs11664336, rs2672591, rs6737767) were excluded, and the remaining 498 SNPs were used for MR analysis. After preliminary sensitivity analyses, 104 grip strength-related SNPs and 99 lumbar spine BMD-related SNPs were included. Four SNPs (rs11664336, rs6737767, rs74265413, rs997850) were excluded, and the remaining 99 SNPs were used for MR analysis. After preliminary sensitivity analyses, 104 grip strength-related SNPs and 99 femoral neck BMD-related SNPs were included. Four SNPs (rs11664336, rs6737767, rs74265413, rs997850) were excluded, and the remaining 95 SNPs were used for MR analysis. All the instrumental variables were strongly correlated (*P* < 5 × 10^–8^ as the screening criterion), indicating that the included instrumental variables were all effective. The bias of weak instrumental variables did not affect the causal inference results of MR analysis.

To investigate the causal relationship between total body BMD and grip strength at different ages, femoral neck BMD and grip strength, and lumbar spine BMD and grip strength, we used reverse Mendelian randomization (MR) analysis. In investigating the causal relationship between total body BMD and grip strength at different age analyses, we selected 60 single-nucleotide polymorphisms (SNP) as instrumental variables to assess the causal effects (Table 1). In investigating the causal relationship between lumbar spine BMD and grip strength, we selected 99 single-nucleotide polymorphisms (SNP) as instrumental variables to assess the causal effects (Table 2). In investigating the causal relationship between femoral neck BMD and grip strength, we selected 99 single-nucleotide polymorphisms (SNP) as instrumental variables to assess the causal effects (Table 3).

**Table 1:** Specific information of SNP (grip strength(right) and total body BMD

SNP	beta.exposure	beta.outcome	pval.outcome	pval.exposure
rs10210654	0.012578	0.0113	0.3545	2.05 E − 08
rs1043515	0.0131519	− 0.0131	0.1795	1.02E − 13
rs10798483	0.0159321	− 0.0038	0.702499	1.84E − 19
rs10799428	− 0.0145885	− 0.0116	0.362	7.39E − 11
rs10846071	− 0.0170039	− 0.0109	0.2696	2.43E − 21
rs11236188	− 0.0169667	0.0041	0.6785	4.72E − 22
rs11243202	0.0126931	0.0097	0.3232	6.04E − 13
rs11664336	0.0124714	− 0.0005	0.9592	1.84E − 12
rs11669079	0.0125683	− 0.0115	0.3004	7.11E − 11
rs11813532	− 0.0101727	0.0058	0.5774	3.94E − 08
rs12127013	− 0.0147955	− 0.0074	0.6239	1.27E − 08
rs12361415	0.0112541	− 0.0299	0.00743995	6.24E − 09
rs12452505	− 0.0143135	− 0.0021	0.8942	1.61E − 08
rs1245463	0.0102737	− 0.002	0.8446	1.20E − 08
rs12562146	0.0143927	− 0.0253	0.06624	8.79E − 09
rs12598856	0.00976282	− 0.0243	0.0176701	4.78E − 08
rs12708450	− 0.0130128	0.0053	0.670101	3.17E − 09
rs12790261	− 0.0259744	0.0018	0.9426	3.12E − 16
rs1280839	0.0104842	0.0093	0.3442	4.65E − 09
rs12914702	0.0117428	− 0.0085	0.570401	3.89E − 09
rs12917449	− 0.0133569	− 0.0063	0.6102	1.58E − 09
rs13017833	− 0.0270737	− 0.0325	0.2234	1.18E − 08
rs13040292	0.0123166	0.0174	0.1317	1.30E − 09
rs13107325	− 0.0307805	0.0307	0.1303	3.07E − 20
rs13150083	− 0.011281	− 0.0221	0.0442996	2.60E − 08
rs1442883	− 0.0111988	− 0.0092	0.4063	3.16E − 08
rs150330307	− 0.0367934	− 0.0107	0.736	1.65E − 13
rs1514665	0.0101011	− 0.0051	0.600999	1.37E − 08
rs1550115	0.0163021	0.0147	0.1874	7.98E − 16
rs1556659	0.0172423	− 0.0231	0.0250202	1.76E − 21
rs17688916	− 0.0145221	− 0.009	0.581201	4.92E − 11
rs1991431	0.0105354	− 0.0309	0.00183101	2.50E − 09
rs200531	0.0130464	− 0.0207	0.0893203	3.86E − 09
rs2194411	0.0149727	0.0043	0.784899	1.75E − 08
rs2265309	− 0.0101519	0.0278	0.00423	7.55E − 09
rs2273555	0.0113403	− 0.0039	0.6947	2.70E − 10
rs2431112	− 0.0105791	− 0.0001	0.9881	2.20E − 09
rs249516	0.0105093	0.0074	0.4592	2.91E − 09
rs2587505	− 0.0102463	0.0018	0.8581	8.55E − 09
rs2672591	− 0.00985699	0.0005	0.9572	2.31E − 08
rs28417075	− 0.0201691	− 0.1124	0.2499	1.35E − 09
rs2854152	0.0134498	0.0013	0.9036	9.62E − 13
rs2871865	− 0.0262029	0.017	0.3052	1.37E − 21
rs2894602	0.0114245	− 0.0043	0.723401	4.26E − 08
rs2971154	− 0.0107366	− 0.0132	0.1954	1.90E − 09
rs3116605	− 0.0189418	− 0.0152	0.2069	5.67E − 19
rs34217742	0.0157725	− 0.003	0.8433	4.10E − 09
rs34530577	− 0.010671	0.0128	0.1943	1.51E − 09
rs34588175	− 0.0215982	− 0.0047	0.720701	3.39E − 19
rs34627176	− 0.0116983	0.0052	0.6936	4.96E − 08
rs35910339	− 0.0126967	0.0058	0.5847	1.58E − 11
rs3771498	0.0149393	0.0035	0.7173	2.08E − 17
rs3773853	0.00987952	0.0102	0.3083	2.25E − 08
rs3790076	− 0.0114892	− 0.0232	0.0183101	9.62E − 11
rs417591	0.0142382	− 0.0133	0.2433	2.83E − 11
rs4308051	0.0162767	0.0028	0.8221	2.91E − 14
rs4326984	− 0.00974231	− 0.0092	0.3972	3.65E − 08
rs4373305	− 0.0105389	− 0.0379	0.000146599	4.74E − 09
rs4380799	− 0.0140479	0.0156	0.3759	1.48E − 12
rs4594848	0.0144498	− 0.0053	0.593999	2.45E − 16
rs4621706	− 0.0101992	0.0016	0.8654	8.81E − 09
rs4730984	0.0117684	0.0081	0.5461	1.01E − 08
rs4751671	0.0107422	0.0109	0.2746	1.30E − 09
rs475390	− 0.0138322	0.0236	0.04622	5.09E − 11
rs4764131	− 0.0174614	− 0.0055	0.5866	4.35E − 22
rs4784329	− 0.0133724	0.0014	0.889	5.35E − 14
rs4785574	− 0.0110304	− 0.0039	0.699301	4.54E − 10
rs4886778	0.0129591	− 0.0174	0.0725805	1.56E − 13
rs4927015	0.0129145	0.0118	0.2396	4.92E − 13
rs4945185	− 0.0101724	− 0.0204	0.04144	2.28E − 08
rs56412116	− 0.0124736	0.0087	0.4762	1.32E − 08
rs57316347	− 0.0108078	− 0.0198	0.0573799	1.18E − 08
rs6006984	0.011258	− 0.0189	0.0871806	9.67E − 09
rs635538	− 0.024008	0.0123	0.477	1.88E − 14
rs6425501	0.0101804	− 0.0163	0.1044	3.08E − 08
rs6539284	0.0120237	0.0181	0.0727294	1.17E − 11
rs6693965	− 0.0164366	− 0.0097	0.4832	4.93E − 10
rs6737767	0.0107049	0.0001	0.9939	1.52E − 09
rs6977081	0.0127019	0.0104	0.3045	1.39E − 11
rs700518	0.0110475	0.0321	0.001012	2.91E − 10
rs7071654	0.015589	− 0.0267	0.0448095	9.48E − 11
rs7206195	− 0.0181117	0.0105	0.4266	2.60E − 15
rs721101	0.0122915	− 0.0115	0.304	4.60E − 10
rs7249081	0.0106797	− 0.0092	0.3526	1.47E − 09
rs73307079	0.0135324	0.0014	0.903	4.55E − 10
rs74265413	0.0123642	0.0188	0.0602005	1.51E − 11
rs75069534	0.0171798	− 0.006	0.7565	3.28E − 09
rs7740107	− 0.0170713	− 0.008	0.4759	8.50E − 18
rs77485342	0.0418193	− 0.0114	0.8405	1.10E − 10
rs7760564	0.0118596	0.0095	0.4063	2.09E − 08
rs7871404	0.0124665	0.0145	0.2287	2.70E − 08
rs7968902	− 0.0125769	− 0.0025	0.797	2.94E − 12
rs8012800	0.0110448	0.0046	0.674	4.32E − 08
rs8055199	− 0.011299	− 0.0002	0.9892	1.21E − 09
rs817316	0.0104545	0.0198	0.0713001	5.99E − 09
rs823130	− 0.0146722	0.0028	0.7769	1.37E − 16
rs9267806	− 0.0185844	− 0.0043	0.7212	3.02E − 20
rs9322822	0.0117649	− 0.0244	0.0188599	3.74E − 10
rs934075	− 0.010986	0.0187	0.0723303	5.54E − 09
rs9396861	− 0.0112138	− 0.0132	0.2169	9.65E − 10
rs973767	0.0136436	− 0.0223	0.0931794	7.97E − 09
rs9847951	− 0.00994632	0.0138	0.1698	2.32E − 08
rs997850	− 0.0102322	− 0.0069	0.4868	1.57E − 08

**Table 2:** Specific Information of SNP (grip strength (right) and lumbar spine BMD

SNP	beta.exposure	beta.outcome	pval.outcome	pval.exposure
rs10210654	0.012578	0.001018	0.92769	2.0514E − 08
rs1043515	0.0131519	0.006352	0.477801	1.02376E − 13
rs10798483	0.0159321	0.001358	0.880126	1.83992E − 19
rs10799428	− 0.0145885	− 0.024287	0.0378416	7.38754E − 11
rs11236188	− 0.0169667	− 0.013583	0.126477	4.71954E − 22
rs11243202	0.0126931	0.02759	0.00204099	6.04088E − 13
rs11664336	0.0124714	0.005627	0.532031	1.84289E − 12
rs11669079	0.0125683	0.016173	0.0944496	7.10722E − 11
rs11813532	− 0.0101727	0.008298	0.378001	3.94348E − 08
rs12127013	− 0.0147955	0.005583	0.6846	1.26745E − 08
rs12361415	0.0112541	0.004929	0.615744	6.23634E − 09
rs12452505	− 0.0143135	0.002073	0.874836	1.60565E − 08
rs1245463	0.0102737	− 0.028998	0.00162401	1.20063E − 08
rs12562146	0.0143927	− 0.016608	0.179691	8.79103E − 09
rs12598856	0.00976282	− 0.01312	0.160318	4.78167E − 08
rs12708450	− 0.0130128	− 0.000035	0.997513	3.17088E − 09
rs12790261	− 0.0259744	0.000782	0.967635	3.12104E − 16
rs1280839	0.0104842	− 0.009075	0.320151	4.65425E − 09
rs12914702	0.0117428	− 0.010755	0.354137	3.89233E − 09
rs12917449	− 0.0133569	− 0.020286	0.0743704	1.58107E − 09
rs13017833	− 0.0270737	− 0.00483	0.840521	1.18021E − 08
rs13040292	0.0123166	0.023463	0.0265693	1.29721E − 09
rs13107325	− 0.0307805	0.024843	0.134161	3.06761E − 20
rs13150083	− 0.011281	− 0.000403	0.968982	2.59896E − 08
rs1442883	− 0.0111988	0.002277	0.826049	3.15697E − 08
rs150330307	− 0.0367934	0.013785	0.600152	1.65348E − 13
rs1514665	0.0101011	0.01366	0.13316	1.36811E − 08
rs1550115	0.0163021	0.017974	0.0833105	7.97811E − 16
rs1556659	0.0172423	0.007361	0.426402	1.76441E − 21
rs17688916	− 0.0145221	− 0.05837	9.41001E − 05	4.91813E − 11
rs1991431	0.0105354	0.003821	0.720747	2.5015E − 09
rs200531	0.0130464	− 0.008981	0.431658	3.85771E − 09
rs2265309	− 0.0101519	− 0.002016	0.823884	7.54918E − 09
rs2431112	− 0.0105791	− 0.009604	0.286432	2.20034E − 09
rs249516	0.0105093	0.004836	0.589076	2.90837E − 09
rs2587505	− 0.0102463	− 0.019454	0.033597	8.54594E − 09
rs28417075	− 0.0201691	− 0.011139	0.722756	1.35316E − 09
rs2854152	0.0134498	0.002487	0.799553	9.62055E − 13
rs2871865	− 0.0262029	− 0.030335	0.0340001	1.37025E − 21
rs2894602	0.0114245	0.002208	0.838328	4.26393E − 08
rs2971154	− 0.0107366	− 0.009072	0.329663	1.90174E − 09
rs3116605	− 0.0189418	− 0.011946	0.272982	5.67414E − 19
rs34217742	0.0157725	0.001164	0.932224	4.09845E − 09
rs34530577	− 0.010671	− 0.005994	0.505548	1.50741E − 09
rs34588175	− 0.0215982	− 0.019764	0.107337	3.38766E − 19
rs34627176	− 0.0116983	− 0.002034	0.859372	4.96215E − 08
rs35910339	− 0.0126967	− 0.002626	0.787876	1.5838E − 11
rs3771498	0.0149393	0.005189	0.561524	2.08113E − 17
rs3773853	0.00987952	− 0.001459	0.870809	2.25113E − 08
rs3790076	− 0.0114892	0.003853	0.669197	9.62277E − 11
rs417591	0.0142382	− 0.023894	0.0261048	2.83074E − 11
rs4308051	0.0162767	0.00029	0.979675	2.91206E − 14
rs4326984	− 0.00974231	0.016126	0.0890923	3.65309E − 08
rs4373305	− 0.0105389	− 0.001234	0.89323	4.74045E − 09
rs4380799	− 0.0140479	− 0.012079	0.354728	1.47877E − 12
rs4594848	0.0144498	0.005005	0.587566	2.45358E − 16
rs4621706	− 0.0101992	− 0.01802	0.0434981	8.81252E − 09
rs4730984	0.0117684	− 0.001674	0.872819	1.01221E − 08
rs4751671	0.0107422	0.009241	0.301922	1.30416E − 09
rs475390	− 0.0138322	0.004979	0.641068	5.08745E − 11
rs4764131	− 0.0174614	− 0.022054	0.0168958	4.35311E − 22
rs4784329	− 0.0133724	0.016948	0.0596705	5.34811E − 14
rs4785574	− 0.0110304	− 0.009956	0.275335	4.53806E − 10
rs4886778	0.0129591	− 0.015792	0.0782132	1.56387E − 13
rs4927015	0.0129145	0.014166	0.118733	4.91926E − 13
rs4945185	− 0.0101724	− 0.009043	0.332146	2.27809E − 08
rs56412116	− 0.0124736	0.028698	0.010931	1.32428E − 08
rs57316347	− 0.0108078	− 0.026045	0.00709006	1.17999E − 08
rs6006984	0.011258	0.007336	0.536421	9.66607E − 09
rs635538	− 0.024008	− 0.021563	0.171012	1.87715E − 14
rs6425501	0.0101804	− 0.00144	0.877304	3.0839E − 08
rs6539284	0.0120237	0.012181	0.181502	1.16923E − 11
rs6693965	− 0.0164366	− 0.041024	0.0017	4.9339E − 10
rs6737767	0.0107049	− 0.011688	0.199287	1.52419E − 09
rs6977081	0.0127019	0.016208	0.0848457	1.39123E − 11
rs700518	0.0110475	0.014743	0.101039	2.91233E − 10
rs7071654	0.015589	0.006045	0.614076	9.482E − 11
rs7206195	− 0.0181117	0.008414	0.478545	2.60136E − 15
rs721101	0.0122915	0.006148	0.545899	4.60013E − 10
rs7249081	0.0106797	0.004858	0.590954	1.4669E − 09
rs73307079	0.0135324	− 0.016861	0.118373	4.55177E − 10
rs74265413	0.0123642	0.002145	0.829357	1.51182E − 11
rs75069534	0.0171798	− 0.004012	0.800035	3.27771E − 09
rs7740107	− 0.0170713	− 0.001052	0.918222	8.49572E − 18
rs77485342	0.0418193	0.01483	0.762994	1.10238E − 10
rs7760564	0.0118596	0.019151	0.0723402	2.0867E − 08
rs7871404	0.0124665	− 0.005041	0.660015	2.69594E − 08
rs7968902	− 0.0125769	0.001954	0.826672	2.939E − 12
rs8012800	0.0110448	− 0.009294	0.366996	4.32046E − 08
rs8055199	− 0.011299	− 0.001073	0.911872	1.21364E − 09
rs817316	0.0104545	− 0.012314	0.199288	5.9888E − 09
rs823130	− 0.0146722	− 0.012263	0.174295	1.37183E − 16
rs9267806	− 0.0185844	0.00723	0.568433	3.01995E − 20
rs9322822	0.0117649	− 0.019921	0.0379586	3.74033E − 10
rs934075	− 0.010986	0.01685	0.0818276	5.5363E − 09
rs9396861	− 0.0112138	− 0.006542	0.493282	9.6545E − 10
rs973767	0.0136436	− 0.015345	0.20512	7.97315E − 09
rs9847951	− 0.00994632	0.000008	0.999255	2.31574E − 08
rs997850	− 0.0102322	− 0.007211	0.428459	1.57348E − 08

**Table 3:** Specific Information of SNP (grip strength (right) and femoral neck BMD

SNP	beta.exposure	beta.outcome	pval.outcome	pval.exposure
rs10210654	0.012578	0.001018	0.92769	2.0514E − 08
rs1043515	0.0131519	0.006352	0.477801	1.02376E − 13
rs10798483	0.0159321	0.001358	0.880126	1.83992E − 19
rs10799428	− 0.0145885	− 0.024287	0.0378416	7.38754E − 11
rs11236188	− 0.0169667	− 0.013583	0.126477	4.71954E − 22
rs11243202	0.0126931	0.02759	0.00204099	6.04088E − 13
rs11664336	0.0124714	0.005627	0.532031	1.84289E − 12
rs11669079	0.0125683	0.016173	0.0944496	7.10722E − 11
rs11813532	− 0.0101727	0.008298	0.378001	3.94348E − 08
rs12127013	− 0.0147955	0.005583	0.6846	1.26745E − 08
rs12361415	0.0112541	0.004929	0.615744	6.23634E − 09
rs12452505	− 0.0143135	0.002073	0.874836	1.60565E − 08
rs1245463	0.0102737	− 0.028998	0.00162401	1.20063E − 08
rs12562146	0.0143927	− 0.016608	0.179691	8.79103E − 09
rs12598856	0.00976282	− 0.01312	0.160318	4.78167E − 08
rs12708450	− 0.0130128	− 0.000035	0.997513	3.17088E − 09
rs12790261	− 0.0259744	0.000782	0.967635	3.12104E − 16
rs1280839	0.0104842	− 0.009075	0.320151	4.65425E − 09
rs12914702	0.0117428	− 0.010755	0.354137	3.89233E − 09
rs12917449	− 0.0133569	− 0.020286	0.0743704	1.58107E − 09
rs13017833	− 0.0270737	− 0.00483	0.840521	1.18021E − 08
rs13040292	0.0123166	0.023463	0.0265693	1.29721E − 09
rs13107325	− 0.0307805	0.024843	0.134161	3.06761E − 20
rs13150083	− 0.011281	− 0.000403	0.968982	2.59896E − 08
rs1442883	− 0.0111988	0.002277	0.826049	3.15697E − 08
rs150330307	− 0.0367934	0.013785	0.600152	1.65348E − 13
rs1514665	0.0101011	0.01366	0.13316	1.36811E − 08
rs1550115	0.0163021	0.017974	0.0833105	7.97811E − 16
rs1556659	0.0172423	0.007361	0.426402	1.76441E − 21
rs17688916	− 0.0145221	− 0.05837	9.41001E − 05	4.91813E − 11
rs1991431	0.0105354	0.003821	0.720747	2.5015E − 09
rs200531	0.0130464	− 0.008981	0.431658	3.85771E − 09
rs2265309	− 0.0101519	− 0.002016	0.823884	7.54918E − 09
rs2431112	− 0.0105791	− 0.009604	0.286432	2.20034E − 09
rs249516	0.0105093	0.004836	0.589076	2.90837E − 09
rs2587505	− 0.0102463	− 0.019454	0.033597	8.54594E − 09
rs28417075	− 0.0201691	− 0.011139	0.722756	1.35316E − 09
rs2854152	0.0134498	0.002487	0.799553	9.62055E − 13
rs2871865	− 0.0262029	− 0.030335	0.0340001	1.37025E − 21
rs2894602	0.0114245	0.002208	0.838328	4.26393E − 08
rs2971154	− 0.0107366	− 0.009072	0.329663	1.90174E − 09
rs3116605	− 0.0189418	− 0.011946	0.272982	5.67414E − 19
rs34217742	0.0157725	0.001164	0.932224	4.09845E − 09
rs34530577	− 0.010671	− 0.005994	0.505548	1.50741E − 09
rs34588175	− 0.0215982	− 0.019764	0.107337	3.38766E − 19
rs34627176	− 0.0116983	− 0.002034	0.859372	4.96215E − 08
rs35910339	− 0.0126967	− 0.002626	0.787876	1.5838E − 11
rs3771498	0.0149393	0.005189	0.561524	2.08113E − 17
rs3773853	0.00987952	− 0.001459	0.870809	2.25113E − 08
rs3790076	− 0.0114892	0.003853	0.669197	9.62277E − 11
rs417591	0.0142382	− 0.023894	0.0261048	2.83074E − 11
rs4308051	0.0162767	0.00029	0.979675	2.91206E − 14
rs4326984	− 0.00974231	0.016126	0.0890923	3.65309E − 08
rs4373305	− 0.0105389	− 0.001234	0.89323	4.74045E − 09
rs4380799	− 0.0140479	− 0.012079	0.354728	1.47877E − 12
rs4594848	0.0144498	0.005005	0.587566	2.45358E − 16
rs4621706	− 0.0101992	− 0.01802	0.0434981	8.81252E − 09
rs4730984	0.0117684	− 0.001674	0.872819	1.01221E − 08
rs4751671	0.0107422	0.009241	0.301922	1.30416E − 09
rs475390	− 0.0138322	0.004979	0.641068	5.08745E − 11
rs4764131	− 0.0174614	− 0.022054	0.0168958	4.35311E − 22
rs4784329	− 0.0133724	0.016948	0.0596705	5.34811E − 14
rs4785574	− 0.0110304	− 0.009956	0.275335	4.53806E − 10
rs4886778	0.0129591	− 0.015792	0.0782132	1.56387E − 13
rs4927015	0.0129145	0.014166	0.118733	4.91926E − 13
rs4945185	− 0.0101724	− 0.009043	0.332146	2.27809E − 08
rs56412116	− 0.0124736	0.028698	0.010931	1.32428E − 08
rs57316347	− 0.0108078	− 0.026045	0.00709006	1.17999E − 08
rs6006984	0.011258	0.007336	0.536421	9.66607E − 09
rs635538	− 0.024008	− 0.021563	0.171012	1.87715E − 14
rs6425501	0.0101804	− 0.00144	0.877304	3.0839E − 08
rs6539284	0.0120237	0.012181	0.181502	1.16923E − 11
rs6693965	− 0.0164366	− 0.041024	0.0017	4.9339E − 10
rs6737767	0.0107049	− 0.011688	0.199287	1.52419E − 09
rs6977081	0.0127019	0.016208	0.0848457	1.39123E − 11
rs700518	0.0110475	0.014743	0.101039	2.91233E − 10
rs7071654	0.015589	0.006045	0.614076	9.482E − 11
rs7206195	− 0.0181117	0.008414	0.478545	2.60136E − 15
rs721101	0.0122915	0.006148	0.545899	4.60013E − 10
rs7249081	0.0106797	0.004858	0.590954	1.4669E − 09
rs73307079	0.0135324	− 0.016861	0.118373	4.55177E − 10
rs74265413	0.0123642	0.002145	0.829357	1.51182E − 11
rs75069534	0.0171798	− 0.004012	0.800035	3.27771E − 09
rs7740107	− 0.0170713	− 0.001052	0.918222	8.49572E − 18
rs77485342	0.0418193	0.01483	0.762994	1.10238E − 10
rs7760564	0.0118596	0.019151	0.0723402	2.0867E − 08
rs7871404	0.0124665	− 0.005041	0.660015	2.69594E − 08
rs7968902	− 0.0125769	0.001954	0.826672	2.939E − 12
rs8012800	0.0110448	− 0.009294	0.366996	4.32046E − 08
rs8055199	− 0.011299	− 0.001073	0.911872	1.21364E − 09
rs817316	0.0104545	− 0.012314	0.199288	5.9888E − 09
rs823130	− 0.0146722	− 0.012263	0.174295	1.37183E − 16
rs9267806	− 0.0185844	0.00723	0.568433	3.01995E − 20
rs9322822	0.0117649	− 0.019921	0.0379586	3.74033E − 10
rs934075	− 0.010986	0.01685	0.0818276	5.5363E − 09
rs9396861	− 0.0112138	− 0.006542	0.493282	9.6545E − 10
rs973767	0.0136436	− 0.015345	0.20512	7.97315E − 09
rs9847951	− 0.00994632	0.000008	0.999255	2.31574E − 08
rs997850	− 0.0102322	− 0.007211	0.428459	1.57348E − 08

### Relationship between grip strength phenotypes in different ages and different parts of body BMD

**Fig. 2:**
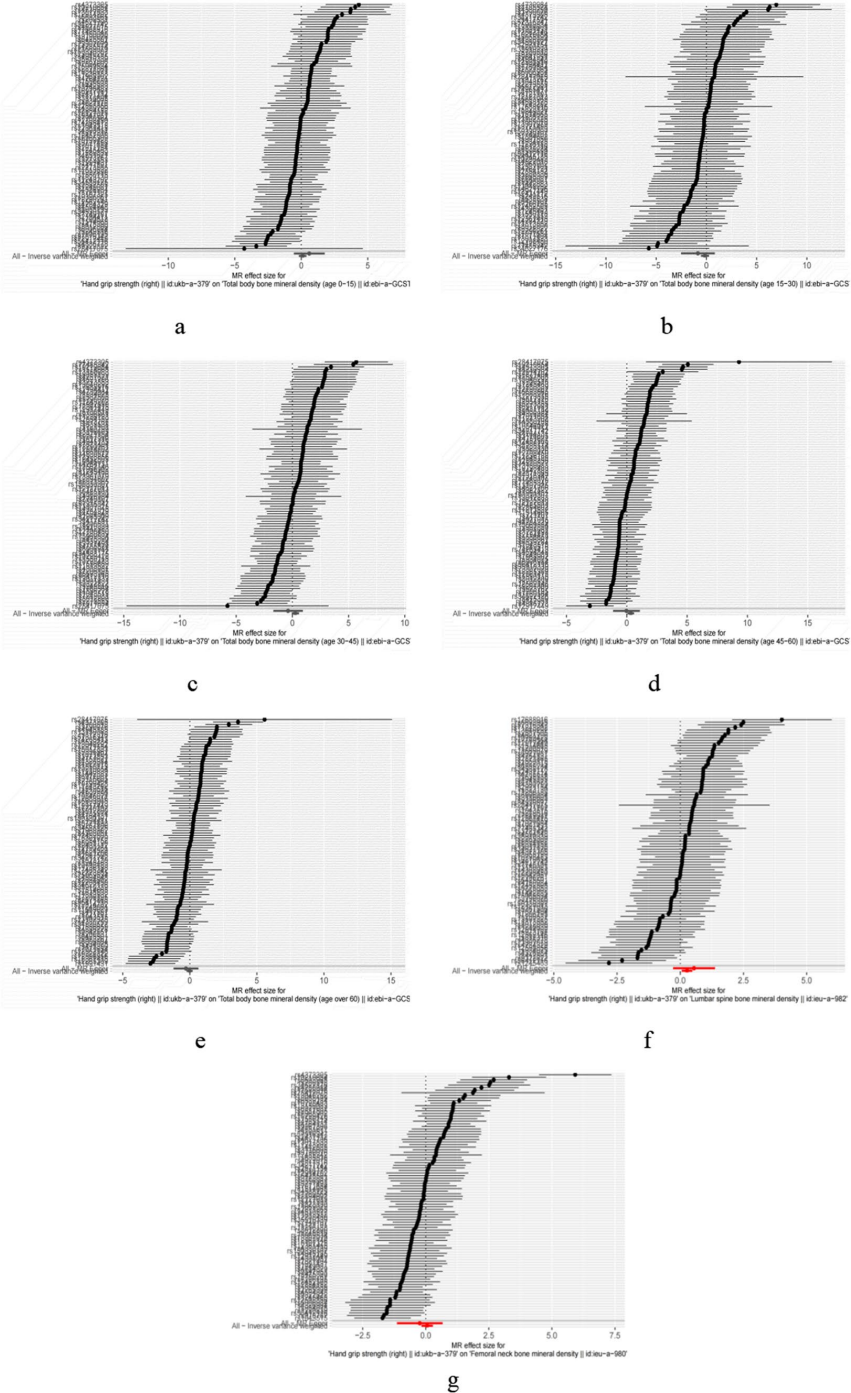
Forest plot of hand grip strength (right) (exposure) and total body BMD of different age, lumbar spine BMD, femoral neck BMD (comeout)

**Fig. 3:**
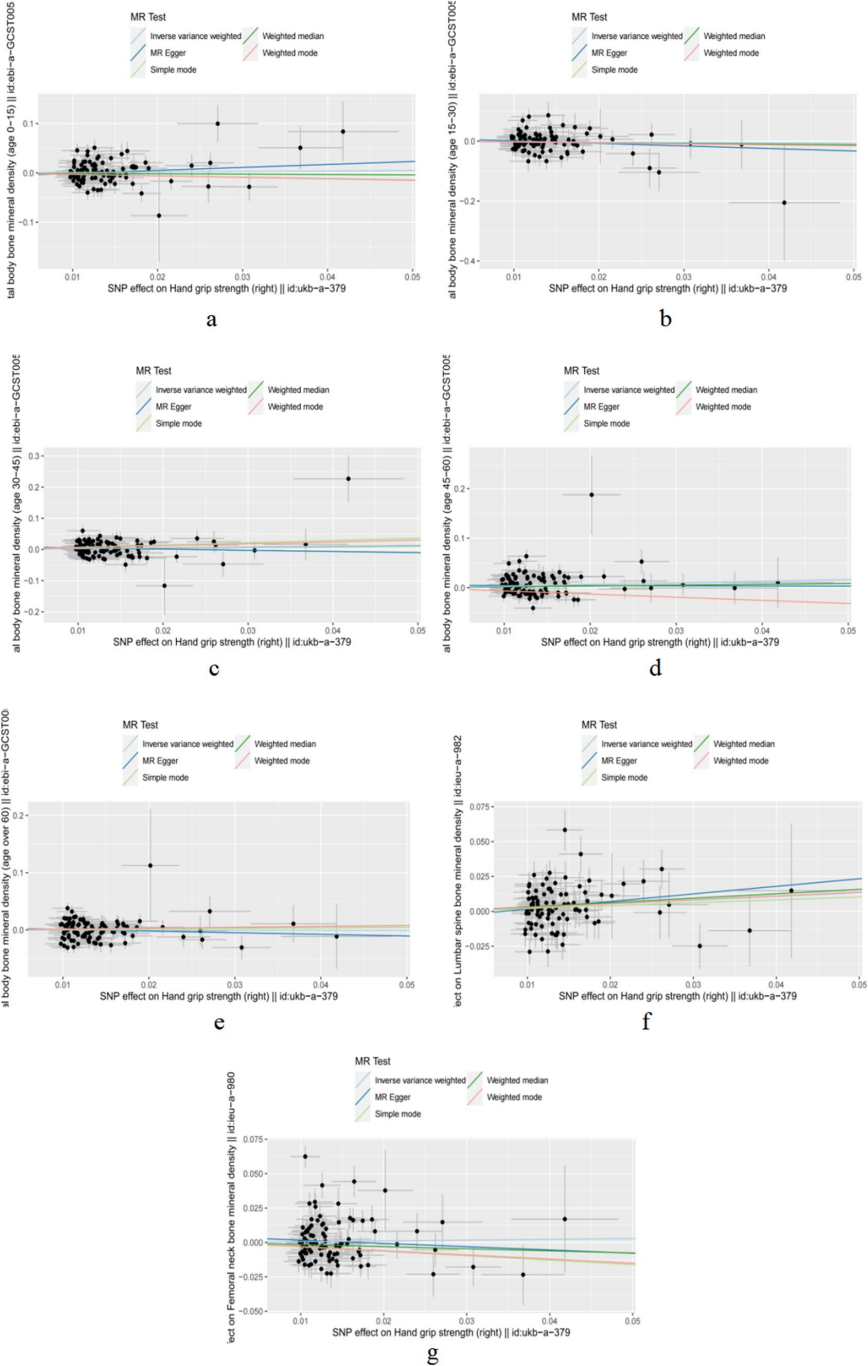
Scatter plot of hand grip strength (right) (exposure) and total body BMD of different age, Lumbar spine BMD, Femoral neck BMD(comeout)

In the primary IVW analyses, hand grip strength was found to be associated with overall total body BMD [OR (95% confidence interval, CI) 1.39 (1.06–1.81), P = 0.017]. Indirectly, hand grip strength was found to be associated with the risk of OP at the age of 45–60. At the other age, hands grip strength was found to have no association with total body BMD or the risk of OP [Total body BMD(AGE:0–15): OR (95% CI) 1.102 (0.83–1.46), P = 0.49; BMD (AGE:15–30): OR (95% CI) 0.86 (0.58–1.28) P = 0.47; Total body BMD (AGE:30–45): OR (95% CI) 1.30 (0.95–1.79), P = 0.10 Total body BMD (AGE > 60) OR (95% CI) 0.99 (0.79–1.22), P = 0.89]. The result is shown in (Table 4, Fig. 2a–e, Fig. 3a–e).

**Table 4:** Result of the relationship between grip strength phenotypes and different ages of total body BMD, lumbar spine BMD, femoral neck BMD

Exposure	Comeout	Label	P	OR	95% CI	Global test	Distortion test
Hand grip strength (right)	Total body bone mineral density (age 0–15)	MR Egger (age 0–15)	0.31	1.82	0.57，5.78	*P*<0.01	*P*=0.01
		Weighted median (age 0–15)	0.65	0.93	0.66，1.30		
		Inverse variance weighted (age 0–15)	0.49	1.10	0.83，1.46		
		Simple mode (age 0–15)	0.54	0.76	0.31，1.86		
		Weighted mode (age 0–15)	0.48	0.74	0.33，1.69		
	Total body bone mineral density (age 15–30)	MR Egger (age 15–30)	0.32	0.43	0.08，2.29		
		Weighted median (age 15–30)	0.42	0.78	0.44，1.41		
		Inverse variance weighted(age 15–30)	0.47	0.86	0.58，1.28		
		Simple mode(age 15–30)	0.65	0.74	0.20，2.70		
		Weighted mode(age 15–30)	0.64	0.74	0.20，2.68		
	Total body bone mineral density (age 30–45)	MR Egger	0.57	0.68	0.18，2.52		
		Weighted median	0.26	1.27	0.84，1.90		
		Inverse variance weighted	0.10	1.30	0.94，1.79		
		Simple mode	0.24	2.05	0.62，6.79		
		Weighted mode	0.30	1.82	0.59，5.57		
	Total body bone mineral density (age 45–60)	MR Egger	0.96	0.97	0.32，2.93		
		Weighted median	0.26	1.19	0.88，1.62		
		Inverse variance weighted	0.02	1.39	1.06，1.81		
		Simple mode	0.16	0.53	0.22，1.28		
		Weighted mode	0.19	0.53	0.21，1.37		
	Total body bone mineral density (age over 60)	MR Egger	0.55	0.75	0.30，1.89		
		Weighted median	0.47	1.10	0.85，1.44		
		Inverse variance weighted	0.89	0.99	0.79，1.23		
		Simple mode	0.80	1.10	0.54，2.25		
		Weighted mode	0.60	1.17	0.65，2.12		
	Lumbar spine bone mineral density	MR Egger	0.20	1.72	0.75, 3.94	*P*<0.01	*P*<0.01
		Weighted median	0.02	1.37	1.07, 1.75		
		Inverse variance weighted	0.01	1.31	1.06, 1.60		
		Simple mode	0.53	1.23	0.66, 2.29		
		Weighted mode	0.34	1.32	0.75, 2.31		
	Femoral neck bone mineral density	MR Egger	0.61	0.79	0.32, 1.94	*P*<0.01	*P*<0.01
		Weighted median	0.16	0.86	0.70, 1.06		
		Inverse variance weighted	0.63	1.06	0.84, 1.32		
		Simple mode	0.29	0.73	0.40, 1.30		
		Weighted mode	0.28	0.74	0.42, 1.28		

Illustration of the Mendelian randomization assumptions with the example of hand grip strength (right) as the exposure risk factor and total body BMD of different age, lumbar spine BMD, femoral neck BMD as the outcome

In the primary IVW analyses, hand grip strength was found to be associated with Lumbar spine BMD [OR(95% confidence interval, CI) 1.31(1.06–1.60), P = 0.01]. Indirectly, hand grip strength was found to be associated with the lumbar spine BMD. However, hand grip strength was not associated with femoral neck BMD. The result is shown in (Table 4, Figs. 2f, g, 3f, g).

In reverse Mendelian randomization analyses, we did not observe any discernible causal relationship between lumbar spine BMD and femoral neck BMD as exposure factors when grip strength was utilized as the outcome variable. We did not observe any discernible causal relationship between total body BMD of different age as exposure factors when grip strength was utilized as the outcome variable (Table 5).

**Table 5:** Result of the relationship between different ages of total body BMD, lumbar spine BMD, femoral neck BMD phenotypes and grip strength (reverse Mendelian randomization analysis)

Exposure	Outcome	Method	*P*	OR	CI	Global test	Distortion test
Total body bone mineral density (age 15–30)	Hand grip strength (right)	Wald ratio	<0.01	0.95	0.93,0.98	P<0.01	P<0.01
Total body bone mineral density (age 0–15)		MR Egger	0.45	0.95	0.84,1.08		
		Weighted median	0.69	1	0.98,1.03		
		Inverse variance weighted	0.39	1.01	0.99,1.04		
		Simple mode	0.24	1.03	0.98,1.08		
		Weighted mode	0.51	1.01	0.98,1.04		
Total body bone mineral density (age 30–45)		MR Egger	0.34	0.94	0.83,1.06		
		Weighted median	0.92	1	0.98,1.02		
		Inverse variance weighted	0.66	1.01	0.98,1.03		
		Simple mode	0.96	1	0.98,1.02		
		Weighted mode	0.9	1	0.98,1.02		
Total body bone mineral density (age over 60)		MR Egger	0.3	0.96	0.89,1.04		
		Weighted median	0.01	1.02	1.00,1.04		
		Inverse variance weighted	0.37	1.01	0.99,1.03		
		Simple mode	0.02	1.04	1.01,1.07		
		Weighted mode	0.03	1.04	1.01,1.07		
Total body bone mineral density (age 45–60)		MR Egger	0.26	0.96	0.90,1.03		
		Weighted median	0.96	1	0.98,1.02		
		Inverse variance weighted	0.57	1.01	0.99,1.03		
		Simple mode	0.39	0.99	0.96,1.02		
		Weighted mode	0.51	0.99	0.97,1.02		
Lumbar spine bone mineral density		MR Egger	0.02	1.94	1.17,3.28	P<0.01	P<0.01
		Weighted median	0.50	1.06	0.90,1.24		
		Inverse variance weighted	0.19	1.08	0.96,1.22		
		Simple mode	0.73	1.05	0.80,1.37		
		Weighted mode	0.70	1.04	0.84,1.30		
Femoral neck bone mineral density		MR Egger	0.62	0.85	0.45，1.61	P<0.01	P<0.01
		Weighted median	0.65	1.04	0.87，1.25		
		Inverse variance weighted	0.38	1.06	0.93，1.20		
		Simple mode	0.62	1.08	0.80，1.46		
		Weighted mode	0.92	1.01	0.78，1.32		

Illustration of the Mendelian randomization assumptions with the example of Total body BMD of different age, lumbar spine BMD, femoral neck BMD as the exposure risk factor and hand grip strength (right) as the outcome

This conclusion is based on the analysis and comprehensive evaluation of data from many relevant studies and, therefore, has high confidence. However, due to the relatively small study sample size, further studies are needed to verify this result in the future. Furthermore, the correlation between BMD and hand grip strength may be influenced by multiple factors, e. g., bone structure, genetic factors, lifestyle, etc., so more in-depth studies are needed to explore the complexity of this issue.

#### Robustness

**Fig. 4:**
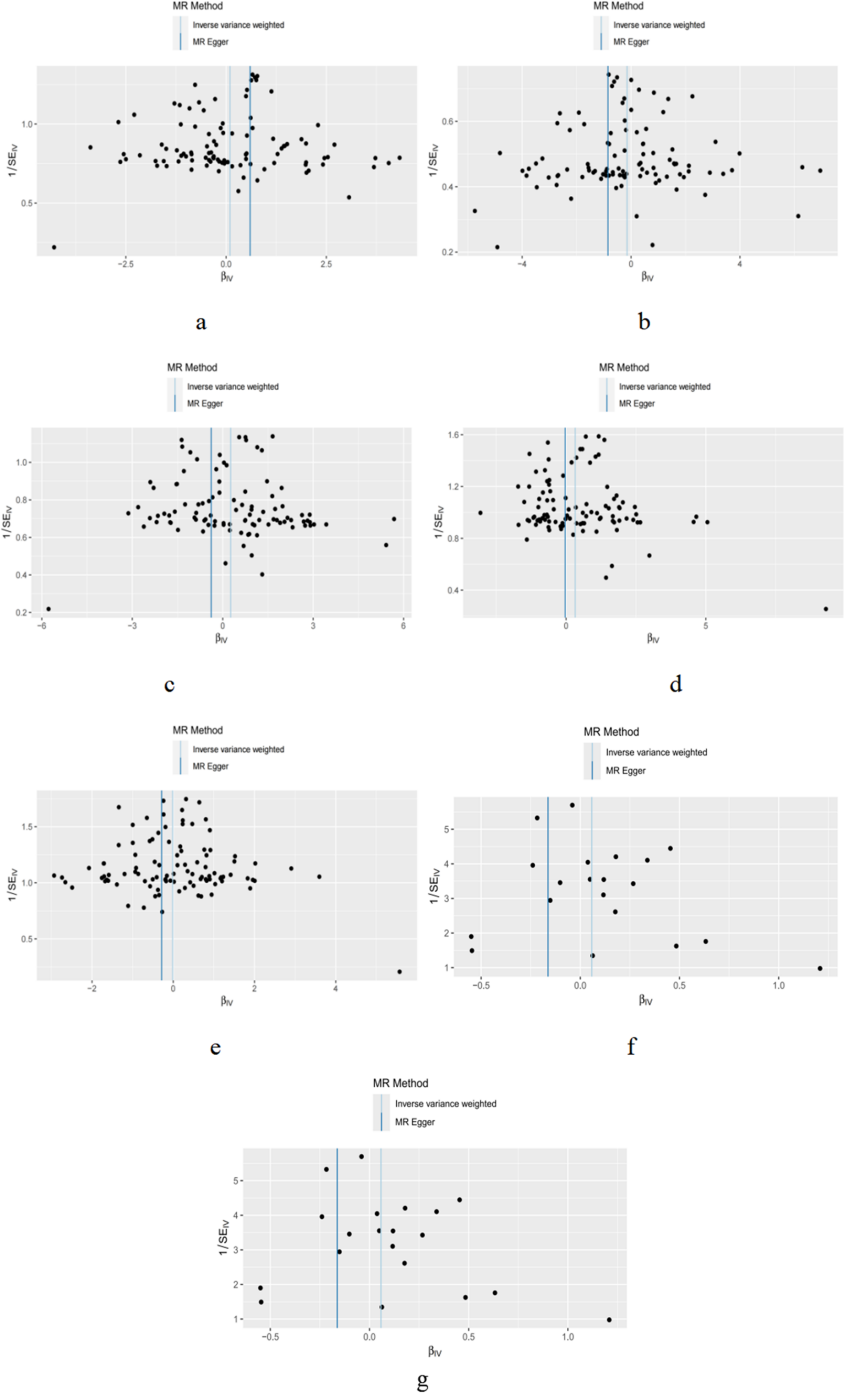
Funnel of hand grip strength (right) (exposure) and total body BMD of different age, lumbar spine BMD, femoral neck BMD (comeout)

MR-Egger [24] regression method tested the possibility of horizontal pleiotropy between SNPs and the results, and the result showed no evidence of horizontal pleiotropy (P > 0.05). The funnel plots suggested that horizontal pleiotropy was not observed in any results (Fig. 4). In addition, the leave-one-out sensitivity analysis plots demonstrated that no single SNP was likely to affect causal association. Therefore, our conclusion was reliable. The MR-PRESSO results showed no outliers of horizontal pleiotropy and possible violations of causal effects in instrumental variables (Global test P < 0.05, Distortion test P = 0.011) in analyzing the relationship between total body BMD and hand grip strength at different ages. The MR-PRESSO [17] results showed that there were no outliers of horizontal pleiotropy and possible violations of causal effects in instrumental variables (Global test P < 0.01, Distortion test P < 0.01) in analyzing the relationship between lumbar spine BMD and hand grip strength. The MR-PRESSO results showed no outliers of horizontal pleiotropy and possible violations of causal effects in instrumental variables (Global test P < 0.01, Distortion test P < 0.01) in analyzing the relationship between femoral neck BMD and hand grip strength. The findings suggested that the null association between genetic predisposition to hand grip strength and total body BMD was not significantly impacted by any SNP.

In the sensitivity analysis, we found that no outliers of horizontal pleiotropy appeared in the MR-PRESSO results (Table 4) indicating that our instrumental variables did not significantly affect the authenticity and reliability of the results. These results suggest that the association between total body BMD of different ages, lumbar spine BMD, femoral neck BMD, and hand grip strength were not significantly affected by a single SNP, leading to reliable conclusions.

## Discussion

This study systematically explores the potential causal relationship between grip strength and BMD from a genetic perspective, using a large population sample across different age groups. Currently, there is a lack of relevant MR studies available in the general population.

According to the molecular mechanism of muscle-related diseases, muscle-secreted factors identified in previous studies are myostatin and interleukin 6. In addition to its negative regulation of skeletal muscle, myostatin has a similar effect on bone mass [27]. When highly expressed in skeletal muscle, IL-6 can positively regulate bone mass, and the mechanism may be through the expression of the RANKL gene [28]. However, the effect of osteocytogenic factors on skeletal muscle was only recently reported. Prostaglandin E2 is a molecule produced in the osteocyte response to shear stress that can increase bone mass. Another new function of prostaglandin E2 was recently found to accelerate cell myogenic differentiation through osteocyte-like medium (PGE (2), sclerostin, and monocyte chemotaxis protein (MCP−3)) [29]. All these studies confirmed the interaction between myokine and bone factors. Recent studies have shown that the muscle-derived signal irisin is the cleaved form of fibronectin type III domain protein 5 (FNDC 5) [30], an exercise-induced myokine, which has been found to improve sarcopenia, grip strength, and muscle mass.

Meanwhile, other studies have found that irisin prevents bone loss and is essential in skeletal remodeling [31, 32]. L-β-aminoisobutyric acid (L-BAIBA) is a small molecule produced by skeletal muscle and animal experiments showed that it can increase grip strength. The results of animal experiments suggest that L-BAIBA exerts a positive regulatory effect on bone synthesis [33]. The receptor activator of nuclear factor-kappa B ligand(RANKL) signal derived from bone, which is produced by vesicles secreted by mature osteoclasts, binds to the RANKL of osteoblasts [34]. This mechanism promotes bone formation through the activation of RANKL reverse signaling, which is utilized in the treatment of osteoporosis. Additionally, it enhances muscle strength. Osteocalcin, a hormone specific to osteoblasts, facilitates mineralization of the extracellular matrix and serves as a marker for osteoblastic bone formation when measured in serum levels [35]. Undercarboxylated osteocalcin in serum independently determines hip BMD in older women [36] and exhibits muscle-protective effects in individuals without metabolic syndrome, particularly among men [37]. Furthermore, osteocalcin stimulates IL-6 synthesis indirectly influencing muscle synthesis [38]. However, the simultaneous or sequential occurrence of these two pathologies remains unclear along with their causal relationship.

According to an epidemiological study of National Health and Nutrition Examination Surveys (NHANES) 2013–2014, handgrip strength can serve as a valuable screening tool for assessing low BMD [39]. A correlation analysis investigating sarcopenia and osteoporosis revealed a significant association between presarcopenic and sarcopenic status with an elevated risk of fracture, indirectly suggesting a positive correlation between grip strength and BMD [40]. However, the current body of research primarily focuses on middle-aged and elderly populations, with limited studies conducted among other age groups. Furthermore, there is a dearth of literature exploring the relationship between handgrip strength and BMD at various anatomical sites. While one study identified a noteworthy link between grip strength and lumbar BMD, no correlation was observed with femoral neck bone density [41]. The underlying mechanism may involve parathyroid hormone (PTH) and osteocalcin, which play crucial roles in maintaining skeletal integrity and muscle growth [42]. Nevertheless, only limited studies have been conducted on this topic thus far; hence, further research is warranted.

Grip strength serves as a reliable measure of patients’ functional capacity and risk of mobility limitations, exhibiting stronger correlations with markers of frailty than chronological age, particularly among older individuals [43]. Both grip strength and bone mass decline positively correlate with advancing age, indicating a gradual deterioration in muscle strength and BMD that contributes to frailty development. Despite conflicting opinions regarding whether grip strength can accurately reflect declines in BMD, current studies demonstrate its significant predictive value for osteoporosis [44]. Due to inconsistent findings in prior studies, the potential influence of other confounding factors on the observed disparity could not be disregarded in the literature review. In this study, stratified Mendelian randomization (MR) analyses were conducted to investigate the association between grip strength and BMD across diverse age cohorts.

Based on the clinical observations, it has been determined that changes in grip strength and bone mass do not occur in the short term; rather, they represent a prolonged clinical process. Analysis of related epidemiological data suggests that the peak age for the decline in grip strength may fall between 40 and 60 years [19, 45]. After the age of 60, grip strength gradually declines. Therefore, positive results are primarily observed between the ages of 45 and 60 when considering grip strength at various age stages as exposure factors, with less significant findings in other age groups. Taking into account previous clinical epidemiological studies, it is speculated that handgrip strength experiences a significant decrease before notable decline in bone density occurs. Research examining the association between sarcopenia and osteoporosis has identified an independent contribution of handgrip strength to BMD and provides an explanation for observed changes in BMD [46].

MR studies use Mendel’s law of genetics and single-nucleotide polymorphisms (SNPs) as instrumental variables (IVs) to infer potential causal relationships between exposure factors and outcomes. This study design minimizes confounding bias and reverse causality while assuming reasonable temporality for causal associations. The results indicate a causal association between grip strength change and whole-body BMD specifically within the 45–60 age group, where grip strength changes are positively correlated with BMD changes. However, no significant association was found among other age groups. Additionally, there is a positive correlation between variations in grip strength and lumbar spine BMD changes but no significant association was identified for femoral neck BMD.

## Limitations

This study systematically assessed the causal effects of grip strength and BMD across different age groups using Mendelian randomization. Although the results suggest an association between grip strength and BMD in the 45–60 age group, there are some limitations: First, only European populations were included as exposed populations and outcomes in this study, while populations of other ethnicities were not analyzed. Previous literature suggests that race may also influence research conclusions. Additionally, the relationship between sarcopenia and BMD has been found to vary based on racial backgrounds, with individuals of African descent showing the strongest correlation followed by those of European descent, and individuals of Asian ancestry having a weaker correlation [47]. An analysis of NHANES 2013–2014 database revealed a positive correlation between elevated handgrip strength and increased BMD even after adjusting for potential confounding factors such as age, gender, race, body mass index (BMI), physical activity level, smoking status, diabetes, hypertension, and high cholesterol [48]. The disparities in handgrip strength among diverse ethnic groups along with variations in the association between grip strength and BMD contribute to discrepancies in final outcomes. While this study aligns with previous research findings overall; given the numerous factors influencing handgrip strength and BMD; additional clinical data encompassing diverse populations from multiple countries is needed to further validate these observations.

Second, this study solely examined the causal relationship between grip strength and BMD within different age groups.

Third, The Mendelian randomization study, as a secondary analysis relying on existing data, must adhere to the original design's age stratification. However, due to its nature as a secondary mining and analysis of literature data, this study cannot conduct subgroup analyses based on gender, race, region, and other factors. This limitation should be acknowledged.

Recognizing these limitations, our subsequent steps involve leveraging clinical data collection and verification. Currently, we are collecting muscle strength and quality data from individuals aged over 30 years to investigate the association between grip strength and bone density. Subsequently, we plan to categorize participants into two groups based on the unique relationship between bone density and age to determine the critical value of grip strength in predicting bone density. This ongoing research is part of our endeavor.

In conclusion, the study's conclusions regarding the relationship between grip strength and BMD in people over 60 years of age have garnered increased attention. This is important due to the rising incidence of osteoporosis in this age group, especially considering that some elderly individuals are bedridden and have limited mobility. Grip strength is a simple test and a valuable predictor of osteoporosis incidence.

Within the age group of 45–60, there was an established causal relationship between grip strength and BMD. Therefore, grip strength can be utilized as one of the screening indicators for predicting osteoporosis in individuals between the ages of 45 and 60. This convenient measure allows for early intervention. However, grip strength was not associated with BMD in other age groups. Consequently, grip strength may not be a reliable predictor of BMD in individuals over 60. Therefore, when assessing the incidence of osteoporosis in individuals above 60, it is necessary to consider other factors such as muscle mass.

According to the conclusion of Mendelian randomization, we will next collect and verify clinical data to further clarify the causal relationship between the development of sarcopenia and the occurrence of osteoporosis to guide the clinical diagnosis and treatment.

## Data Availability

The data supporting this study's findings are available on request from the corresponding author.
